# Disclosing the Novel Protective Mechanisms of Ocrelizumab in Multiple Sclerosis: The Role of PKC Beta and Its Down-Stream Targets

**DOI:** 10.3390/ijms25168923

**Published:** 2024-08-16

**Authors:** Lucrezia Irene Maria Campagnoli, Lara Ahmad, Nicoletta Marchesi, Giacomo Greco, Federica Boschi, Francesco Masi, Giulia Mallucci, Roberto Bergamaschi, Elena Colombo, Alessia Pascale

**Affiliations:** 1 Department of Drug Sciences, Section of Pharmacology, University of Pavia, 27100 Pavia, Italy; lucreziairenem.campagnoli01@universitadipavia.it (L.I.M.C.); nicoletta.marchesi@unipv.it (N.M.); federica.boschi@unipv.it (F.B.); 2 Multiple Sclerosis Center, IRCCS Mondino Foundation, 27100 Pavia, Italy; lara.ahmad@mondino.it (L.A.); giacomo.greco@mondino.it (G.G.); francesco.masi01@universitadipavia.it (F.M.); roberto.bergamaschi@mondino.it (R.B.); elena.colombo@mondino.it (E.C.); 3 Department of Brain and Behavioural Sciences, University of Pavia, 27100 Pavia, Italy; 4 Neurocenter of Southern Switzerland, Ente Ospedaliero Cantonale (EOC), 6900 Lugano, Switzerland

**Keywords:** multiple sclerosis, ocrelizumab, protein kinase C, HIF-1α, VEGF, HuR, MnSOD, HSP70

## Abstract

Ocrelizumab (OCR) is a humanized anti-CD20 monoclonal antibody approved for both Relapsing and Primary Progressive forms of Multiple Sclerosis (MS) treatment. OCR is postulated to act via rapid B cell depletion; however, by analogy with other anti-CD20 agents, additional effects can be envisaged, such as on Protein Kinase C (PKC). Hence, this work aims to explore novel potential mechanisms of action of OCR in peripheral blood mononuclear cells from MS patients before and after 12 months of OCR treatment. We first assessed, up-stream, PKCβII and subsequently explored two down-stream pathways: hypoxia-inducible factor 1 alpha (HIF-1α)/vascular endothelial growth factor (VEGF), and human antigen R (HuR)/manganese-dependent superoxide dismutase (MnSOD) and heat shock proteins 70 (HSP70). At baseline, higher levels of PKCβII, HIF-1α, and VEGF were found in MS patients compared to healthy controls (HC); interestingly, the overexpression of this inflammatory cascade was counteracted by OCR treatment. Conversely, at baseline, the content of HuR, MnSOD, and HSP70 was significantly lower in MS patients compared to HC, while OCR administration induced the up-regulation of these neuroprotective pathways. These results enable us to disclose the dual positive action of OCR: anti-inflammatory and neuroprotective. Therefore, in addition to B cell depletion, the effect of OCR on these molecular cascades can contribute to counteracting disease progression.

## 1. Introduction

Multiple sclerosis (MS) is a complex autoimmune and neurodegenerative disease of the central nervous system (CNS) characterized by demyelinating inflammatory lesions and irreversible axonal injury [1]. To date, disease-modifying therapies have proved successful in slowing disease progression by reducing the inflammatory activity related to relapses, but the control of progression independent of relapses is still an issue [2,3].

Evidence of the involvement of B cells in disease pathophysiology via, among others, the activation of T cells and the formation of lymphatic ectopic aggregates inside the CNS, has led to the use of B cell-depleting monoclonal antibodies in the treatment of MS with both anti-inflammatory and neuroprotective effects [4,5,6].

Ocrelizumab (OCR) is a humanized monoclonal anti-CD20 antibody that was approved by the Food and Drug Administration in 2017 and by the European Medicines Agency in 2018 for the treatment of adult patients with Relapsing Multiple Sclerosis (RMS). In addition, being endowed with neuroprotective effects [7,8], OCR is the first drug ever approved for the early treatment of Primary Progressive MS (PPMS) [8]. Currently, OCR is postulated to mainly act via the rapid depletion of B cells expressing CD20 [9], which in turn, most likely, causes long-lasting changes in the profile of B cells, such as a reduction in their pro-inflammatory phenotype, as demonstrated with other anti-CD20 antibodies [10].

Although its functions are not yet well defined, CD20 is involved in the regulation of B cell growth, activation, and differentiation, together with transmembrane calcium flux [11]. Within this context, by binding CD20, OCR exerts an immunomodulating action; indeed, it causes a rapid depletion of circulating immature and mature B cells, which are implicated in the secretion of pro-inflammatory cytokines, the activation of pro-inflammatory T cells, and the production of auto-antibodies directed against myelin [4].

Of interest, previous studies on other anti-CD20 drugs, mainly rituximab, have shown that anti-CD20 agents can also exert considerable effects on additional intracellular signaling pathways, such as those involving serine/threonine and tyrosine kinases, as well as c-myc [11,12]. Considering the similarities in terms of pharmacokinetics and pharmacodynamics between OCR and rituximab, it can be hypothesized that the efficacy of OCR in delaying disease progression and in promoting neuroprotection might also stem from such intracellular effects. Specifically, among the various intracellular effects implicated in cellular survival and proliferation, the stimulation of the Protein Kinase C (PKC) pathway is of potential significance [12,13]. PKC is a family of at least 10 serine–threonine kinases that are implicated in the course of various pathological processes [14,15,16] and are able to activate different proteins, including the hypoxia-inducible factor 1 (HIF-1) and the human antigen R (HuR) [17,18].

The heterodimeric protein HIF-1 (consisting of an inducible oxygen-sensitive α subunit and a β subunit constitutively expressed) is involved in the regulation of the transcription of many genes following low-oxygen conditions, including *VEGF* [19]. In this regard, the hypoxic condition favors a rise in the expression levels of the HIF-1α subunit (by inhibiting its rapid ubiquitination and subsequent degradation by the proteasome), which in turn binds to the hypoxic response element located within the gene promoter region, thus fostering the transcription of the *VEGF* gene [20]. The vascular endothelial growth factor A (VEGF-A) (often simply called VEGF) protein is an angiogenic factor taking part in key physiological processes such as angiogenesis, vasculogenesis, endothelial proliferation, and the cellular response toward hypoxia [21]. However, it can also play an important role in the development or progression of neurological disorders, such as MS. In this respect, some evidence suggests its deleterious role in the early phase of the disease, where it acts as a pro-inflammatory factor mediating CNS injury and promoting vascular hyperpermeability, finally leading to blood–brain barrier (BBB) disruption, probably one of the first pathogenic mechanisms involved in the development of the inflammatory demyelinating lesions typical in MS [22,23,24].

Concerning the ubiquitous protein HuR, a RNA-binding protein belonging to the ELAV family, it mainly operates as a positive regulator of gene expression and is able to influence every aspect of the post-synthesis fate of the targeted transcripts, with stability and translation being the most relevant ones [25]. Following intra- and extracellular inputs, HuR can act as a regulator of many cellular functions, including proliferation, survival, and apoptosis, as well as immune responses, oxidative stress (OS), and inflammatory processes [26].

Notably, the inflammatory process, a typical feature of this disease, is well known to be tightly linked to OS. Indeed, during early inflammation, the immune cells release reactive oxygen species (ROS), which contribute to the onset of an intracellular signaling pathway that, in turn, leads to the increased gene expression of pro-inflammatory mediators [27,28]. Normally, the body is equipped with endogenous antioxidant defenses capable of neutralizing ROS and protecting the cells from structural and functional damage [29]. For instance, superoxide dismutase 2 (SOD2), also known as manganese-dependent superoxide dismutase (MnSOD), is a mitochondrial antioxidant enzyme able to detoxify the cells from ROS [30]. Additionally, in the presence of OS, the defensive role of the heat shock proteins 70 (HSP70) family has been recognized as well [31,32]. This highly conserved family consists of intracellular proteins that are able to prevent proteins misfolding and aggregating through their chaperone activity and protecting CNS resident cells from apoptosis, and of extracellular proteins able to promote the activation of immune responses [33,34].

Of interest for us, the HuR protein, via distinct *cis*-acting elements called ARE (adenine-uracil-rich elements), is able to specifically bind MnSOD and HSP70 transcripts, positively affecting the expression of their relative proteins [31,35,36]. Furthermore, we previously documented lower levels of HuR in peripheral blood mononuclear cells (PBMCs) from MS patients compared to healthy controls (HC), thus emphasizing the potential involvement of HuR in MS pathogenesis [36,37].

In this study, we aim to explore the novel potential mechanisms of action of OCR in PBMCs from RMS patients. Specifically, we will first assess, before and after treatment up-stream, the PKCβ levels and subsequently explore two down-stream pathways: HIF-1α/VEGF, in order to investigate the possible effect of OCR on inflammation and HuR/MnSOD, and HSP70, in order to evaluate the antioxidant action of OCR. The disease activity during OCR treatment will also be examined in the study.

## 2. Results

### 2.1. Demographic and Clinical Characteristics of MS Patients and HC

As reported in Table 1, we included 17 RRMS patients (6 males, 11 females, mean age 42.17 ± 8.44 years) and age- and sex-matched healthy controls (7 males, 10 females, mean age 42.00 ± 9.26 years). In the MS cohort, the average disease duration was 10 years (range 1–35). The median annualized relapse rate (ARR) was 0.5 (range 0.12–1) at T0. Four patients were naïve to disease-modifying treatments (DMTs); 11 were under treatment with high-efficacy therapy (natalizumab, n = 10, fingolimod n = 1) and two with platform therapies (Interferon beta, n = 2). The reason for the therapeutic switch to OCR was safety (n = 9) or disease activity (n = 4). The median EDSS at inclusion was 2.79 (range 1–6.5); a similar value was observed at T12 (2.61 with range 1–6.5). The median MSSS at inclusion was 4.24 ± 3.07, with a minimal reduction after one year of treatment (3.62 ± 2.88). The patients had few comorbidities, but these included migraines, radiculopathies, and autoimmune thyroiditis. Most patients presented a high brain lesion load (>9; 80%) and spinal cord involvement (77%) at the onset of the disease. No patient had radiological evidence of disease activity at T12.

During OCR exposure, no serious adverse events (SEAs) or adverse events of special interest were observed. Four patients presented infusion-related reactions that were not serious at the first administration of OCR.

The lymphocyte count was normal at T0 in all patients; one patient presented lymphocytopenia grade 1 before the third infusion of OCR (lymphocyte count 800/µL). One patient reported an infection of the urinary tract of mild severity that was solved with antibiotic assumption.

### 2.2. Effect of Ocrelizumab Treatment on the PKCβII/HIF-1α/VEGF Inflammatory Cascade in PBMCs

**Figure 1:**
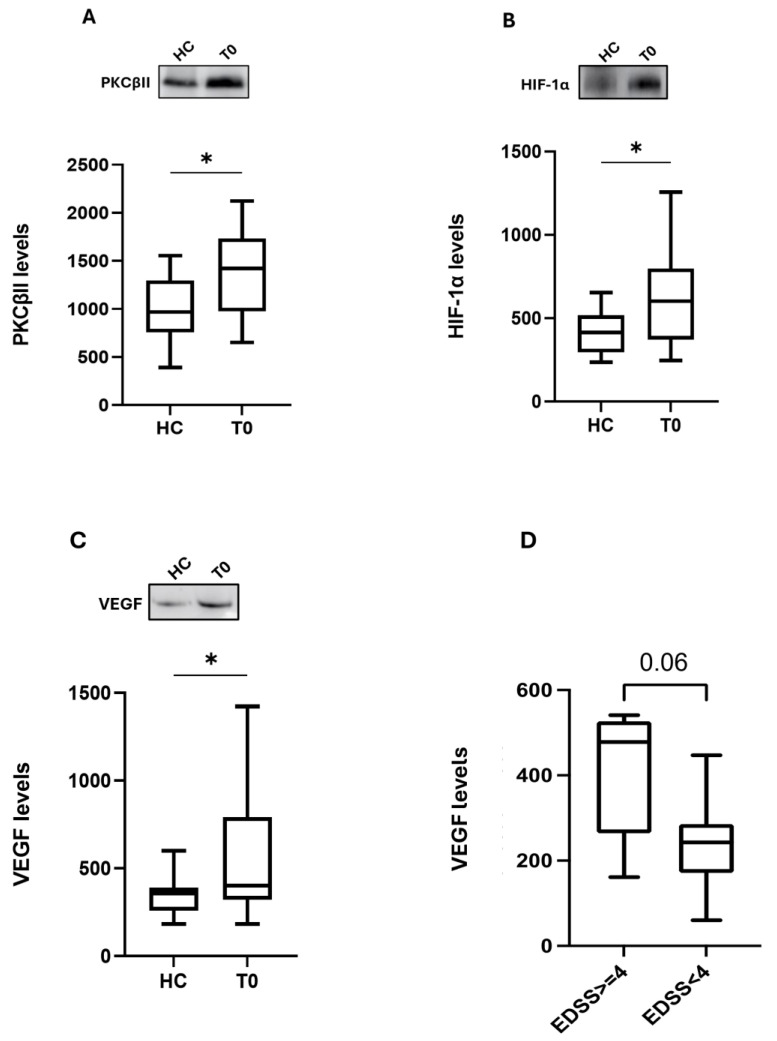
PKCβII, HIF-1α, and VEGF basal protein expression in PBMCs from HC and MS patients. Upper side: cropped representative western blotting images; lower side: boxplots of PKCβII (**A**), HIF-1α (**B**), and VEGF (**C**) protein expression in PBMCs from HC (n = 17) and MS patients (T0, n = 17), and from MS patients in relation to baseline EDSS scores (**D**). The results are expressed as the mean grey level ratios × 10^3^ (mean ± S.E.M.) of the PKCβII (**A**), HIF-1α (**B**), and VEGF (**C**,**D**) immunoreactivities measured by western blotting and normalized on ponceau signals. Data were analyzed by the Mann–Whitney test; * *p* < 0.05. HC = healthy controls; T0 = MS patients before OCR (ocrelizumab) therapy; EDSS: Expanded Disability Status Scale.

Given that, as mentioned, PKC represents a potential target of OCR and HIF-1α is one of the proteins activated by it, which in turn induces the production of VEGF, we first investigated the possible action of OCR on this inflammatory cascade, studying the protein content of PKCβII, HIF-1α, and VEGF in PBMCs from MS patients and HC. At baseline, before OCR exposure, the western blotting analysis showed a significant difference between HC (n = 17) and MS patients (T0, n = 17). In detail, MS patients (T0) expressed higher levels of PKCβII (median 1422; IQ: 974–1734 vs. median 967; IQ: 758–1297, *p* < 0.05), HIF-1α (median 602; IQ: 371–798 vs. median 413; IQ: 294–518, *p* < 0.05), and VEGF (median 401; IQ: 321–794 vs. median 357; IQ: 260–389, *p* < 0.05) compared to the HC group (Figure 1A–C). No significant correlations between the basal PKCβII, HIF-1α, and VEGF values and sex, age, MSSS scores, ARR, previous treatment, or disease duration were observed. However, a relationship, although not reaching statistical significance, was observed between the basal VEGF levels and EDSS; indeed, patients with a higher level of disability (EDSS > 4) displayed higher VEGF levels (411.8 vs. 239.6, *p* = 0.06) (Figure 1D). This last result emphasizes the key role played by VEGF in the pathogenesis of MS and its course and severity.

**Figure 2:**
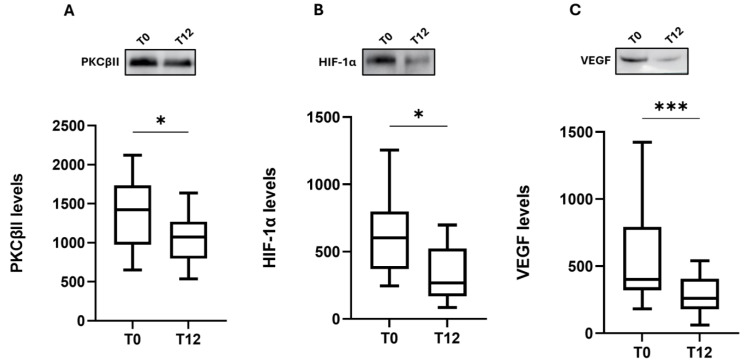
Effect of ocrelizumab on the PKCβII/HIF-1α/VEGF cascade. Upper side: cropped representative western blotting images; lower side: boxplots of PKCβII (**A**), HIF-1α (**B**), and VEGF (**C**) protein expression in PBMCs at baseline (T0) and after 12 months of OCR treatment (T12, n = 17). The results are expressed as the mean grey level ratios × 10^3^ (mean ± S.E.M.) of the PKCβII (**A**), HIF-1α (**B**), and VEGF (**C**) immunoreactivities measured by western blotting and normalized on ponceau signals. Data were analyzed by the Wilcoxon signed-rank test; * *p* < 0.05, *** *p* < 0.001. T0 = MS patients before OCR (ocrelizumab) therapy, T12 = MS patients after 12 months of OCR treatment.

Of interest, we found that the overexpression of this inflammatory cascade was counteracted by OCR treatment; indeed, after 12 months of drug administration (T12, n = 17), the content of these three proteins showed a significant reduction (Figure 2; PKCβII: median 1071; IQ: 796–1271 vs. median 1422; IQ: 974–1734, *p* < 0.05; HIF-1α: median 268; IQ: 168–524 vs. median 602; IQ: 371–798, *p* < 0.05; VEGF: median 261; IQ: 178–407 vs. median 401; IQ: 321–794, *p* < 0.001), exhibiting values similar to those of HC samples (Figure 4A–C).

### 2.3. Effect of Ocrelizumab Treatment on the HuR, MnSOD, and HSP70 Antioxidant Pathways in PBMCs

Since HuR, as previously reported, is another target of PKCβII and can positively affect the expression of its down-stream targets MnSOD and HSP70 [36,37], we then quantified the protein content of these three proteins to investigate the possibility that OCR may be also endowed with antioxidant action.

**Figure 3:**
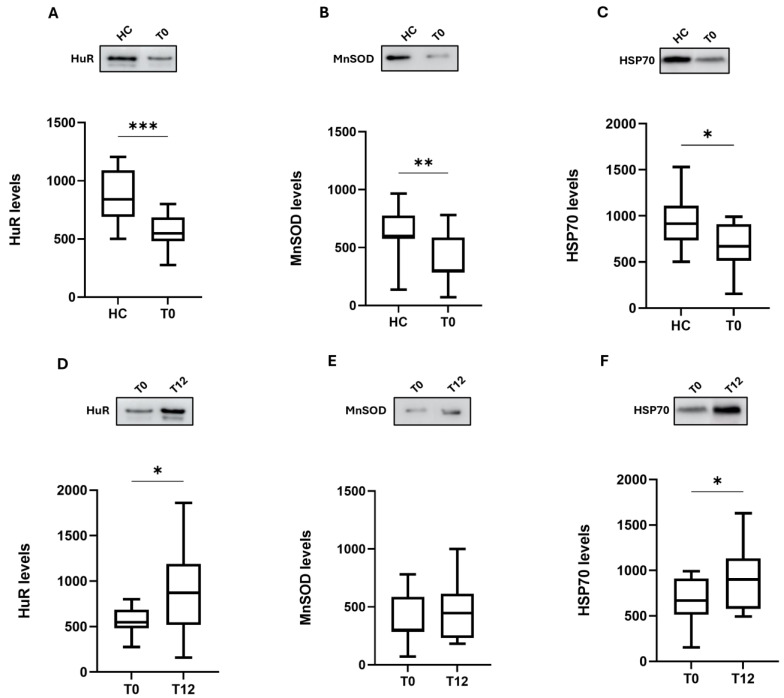
Effect of ocrelizumab on the HuR, MnSOD, and HSP70 neuroprotective cascades in MS patients. Upper side: cropped representative western blotting images; lower side: boxplots of HuR (**A**,**D**), MnSOD (**B**,**E**), and HSP70 (**C**,**F**) protein expression in PBMCs from HC (n = 17) and MS patients (T0: n = 17 and T12: n = 17). The results are expressed as the mean grey level ratios × 10^3^ (mean ± S.E.M.) of the HuR, MnSOD, and HSP70 immunoreactivities measured by western blotting and normalized on ponceau signals. Data were analyzed by the Mann–Whitney test for independent samples (HC-T0) and the Wilcoxon signed-rank test for paired samples (T0 and T12); * *p* < 0.05, ** *p* < 0.01, *** *p* < 0.001. HC = healthy controls; T0 = MS patients before OCR (ocrelizumab) therapy; T12 = MS patients after 12 months of OCR treatment.

We first analyzed the levels of these proteins in MS patients at baseline. As can be seen in Figure 3, at baseline (T0), these proteins are down-regulated. Indeed, the western blotting analysis revealed a significantly lower protein content of HuR (median 548; IQ: 480–686 vs. median 842; IQ: 691–1091, *p* < 0.001), MnSOD (median 300; IQ: 283–587 vs. median 599; IQ: 576–775, *p* < 0.01), and HSP70 (median 669; IQ: 513–911 vs. median 915; IQ: 734–1110, *p* < 0.05) in MS patients compared to HC (Figure 3A–C). Therefore, these findings highlight that these neuroprotective mechanisms are less evident in MS patients.

Of note, after 12 months of OCR administration, we observed an up-regulation of these neuroprotective pathways; in fact, the protein expression of HuR (median 871; IQ: 517–1189 vs. median 548; IQ: 480–686, *p* < 0.05) and HSP70 (median 901; IQ: 577–1132 vs. median 669; IQ: 513–911, *p* < 0.05) was significantly elevated with respect to the baseline (T0), reaching the levels observed in HC subjects. Regarding MnSOD expression, a slight rise, although not significant, was detected at T12 (median 445; IQ: 231–613 vs. median 300; IQ: 283–587, *p* = 0.24) (Figure 3D–F).

No significant correlations between the basal HuR, MnSOD, and HSP70 values and sex, age, EDSS scores, ARR, treatment or disease duration were observed at baseline or after OCR treatment.

**Figure 4:**
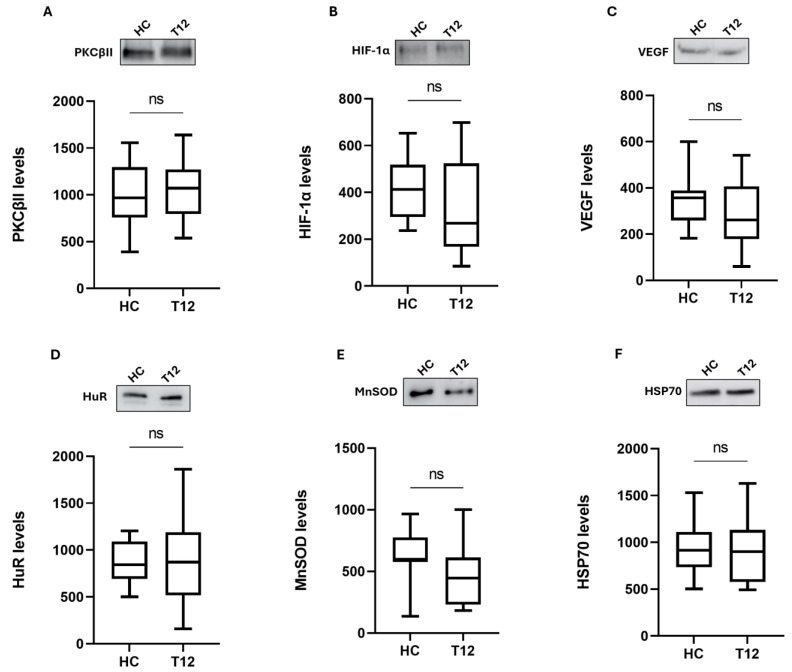
Comparison of protein expression between healthy controls and MS patients after 12 months of ocrelizumab treatment. Boxplots of PKCβII (**A**), HIF-1α (**B**), VEGF (**C**), HuR (**D**), MnSOD (**E**), and HSP70 (**F**) protein expression in PBMCs from HC (n = 17) and MS patients (T12, n = 17). The results are expressed as the mean grey level ratios × 10^3^ (mean ± S.E.M.) of the PKCβII, HIF-1α, VEGF, HuR, MnSOD, and HSP70 immunoreactivities measured by western blotting and normalized on ponceau signals. Data were analyzed by the Mann–Whitney test for independent samples (HC-T12); ns = not significant. HC = healthy controls; T12 = MS patients after 12 months of ocrelizumab (OCR) treatment.

After 12 months of OCR treatment, the levels of all the investigated proteins were comparable to those of the healthy controls (Figure 4D–F).

## 3. Discussion

As reported above, OCR is a high-efficacy disease-modifying treatment approved for RMS and the first drug ever approved for the early treatment of PPMS with evidence of inflammatory activity. Indeed, this drug has undeniable anti-inflammatory action and has shown neuroprotective effects [7,8]. Nonetheless, the molecular mechanisms underlying this double role are not yet fully understood. Considering the structural, pharmacokinetic, and pharmacodynamic similarities between OCR and rituximab, it can be hypothesized that the neuroprotective effect of OCR might also stem from similar intracellular effects [11,12]. Therefore, we aimed to disclose the functioning of OCR by studying novel intracellular signalling pathways, starting from the PKCβ/HIF-1α/VEGF cascade in PBMCs from RMS patients before OCR therapy, and after 12 months of ongoing treatment. Thereafter, we also focused our attention on the neuroprotective pathways involving HuR, MnSOD, and HSP70.

We observed that the PKCβ levels were higher in MS patients (T0) compared to HC; indeed, this finding is in agreement with data in the literature documenting that PKCβ is induced under inflammatory conditions [38]. Our data also show that OCR seems to be able to counteract this aberrant increase in PKCβ expression after 12 months of treatment. The impact of the inhibition of PKCβ expression on CNS diseases is still poorly understood. Nevertheless, it was demonstrated that its inhibition in endothelial cells reduces vascular permeability via the regulation of tight junction protein expression, while in the CNS, it prevents T cells infiltration through the attenuation of vascular permeability and ameliorates disease activity in the experimental autoimmune encephalomyelitis (EAE) animal model [39,40,41].

A similar trend of reduction was detected for HIF-1α, which acts as a transcription factor for VEGF mRNA under hypoxic conditions and whose upregulation correlates with *VEGF* gene activation and the initiation of angiogenesis [42,43].

HIF-1α’s role in MS is only partially known; for instance, histopathological studies have found, in active lesions with distal dying-back oligodendrogliopathy, the pronounced expression of HIF-1α, suggesting that hypoxic tissue damage plays a role in specific demyelinating patterns. HIF-1α is also slightly increased in EAE mice [44,45].

VEGF is a signaling molecule involved in critical processes such as physiological and pathological angiogenesis, and the cellular response to hypoxia. A growing body of evidence suggests that VEGF may play a significant harmful role in the relapsing stage of MS, for example, by increasing BBB permeability [22]. Notably, VEGF primarily promotes vascular hyperpermeability by phosphorylating endothelial tight junction proteins, thus modulating their degradation and ultimately leading to BBB disruption [46,47]. Additionally, VEGF regulates vessel growth and acts as a chemoattractant for monocytes and lymphocytes, thereby promoting neuroinflammation [48]. Interestingly, a remarkable increase in VEGF levels has been observed in mice during the acute phase of murine MOG-induced MS, which correlates with the clinical score [44], as well as in MS patients during clinical disease relapses [49].

In line with this evidence, we observed a higher content of VEGF in MS patients at baseline (T0) compared to HC. In addition, patients with a higher level of disability (EDSS > 4) presented a more elevated expression of this protein. Our study, for the first time, documents the positive impact of the administration of an MS drug, namely OCR, on the VEGF profile. Indeed, a significant reduction in its protein expression was noticed after 12 months of OCR treatment and, interestingly, the VEGF content decreased to the level observed in HC individuals, similar to the PKCβ profile. As already proposed by others, VEGF could be potentially used as a prognostic and treatment response biomarker of MS, but it is unlikely that it could be considered a specific marker for the disease [50]. In fact, as an angiogenic factor, VEGF is known to be involved in other neurological diseases of the CNS, particularly those that are cerebrovascular in nature [51].

Concerning the HuR protein, we observed an opposite trend with respect to VEGF. This finding, though, is in line with our previous results obtained with another drug employed for MS, namely dimethyl fumarate [36,37].

These data encouraged us to explore two down-stream targets of HuR, whose role in neurodegenerative disorders has been described [30,31,32], that may show neuroprotective action through their antioxidant function: MnSOD and HSP70. The obtained findings show that, at baseline, both proteins are significantly decreased in MS patients and that OCR treatment is able to restore the levels observed in the controls. Accordingly, in the context of neurodegenerative disorders, MnSOD has been reported as an enzyme capable of detoxifying cells from oxidative stress [30]. Moreover, we previously demonstrated that its transcript is targeted by HuR, and that the relative protein can contribute to counteracting MS progression [36].

In regard to HSP70, it is in fact known that this protein provides neuroprotection from cerebral infarction [52,53]. In addition, with reference to MS, it has been proposed that, when released by glial cells, HSP70 may protect neurons from inflammatory and neurodegenerative processes and tissues from demyelination, as well as contribute to myelin repair [53]. In agreement with this concept, the overexpression of most HSPs (including HSP70) has been found in the CNS lesions of MS patients and in the EAE animal model [54].

Overall, it is tempting to hypothesize that OCR, while counteracting a decrease in HuR, might also favor its binding towards “beneficial” targets, such as MnSOD and HSP70, as we previously documented for dimethyl fumarate [36]. Nonetheless, further hypotheses might be formulated following a longer observation period.

The main limitations of the present study are related to the small sample size and the limited observation period, with the need to expand our research over a longer follow-up period and a wider population, including a focus on the primary progressive phenotype. Moreover, each analysis was conducted on peripheral PBMCs, giving us hints as to what happens only outside the CNS.

**Figure 5:**
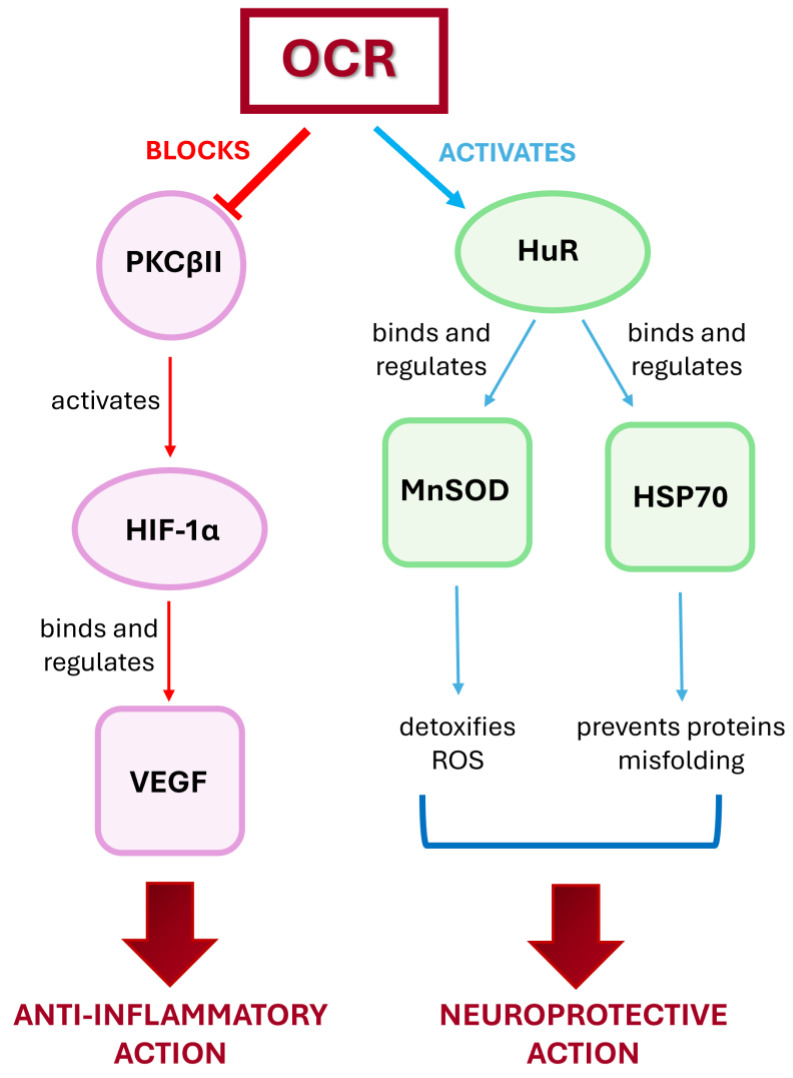
Synthetic representation of the two proposed novel mechanisms of action of ocrelizumab: anti-inflammatory and neuroprotective. See text for details.

Nevertheless, despite these limitations, taken together, these results allow us to demonstrate the dual novel positive action of OCR, in addition to B cell depletion. Indeed, on the one hand, OCR is able to hinder the harmful effect of VEGF by turning off the PKCβII/HIF-1α cascade; on the other hand, it triggers two neuroprotective pathways via MnSOD and HSP70, sustained by HuR (Figure 5). The activation of these molecular cascades can thus represent an additional weapon that the cells can exploit to counteract the progression of this disabling disease.

## 4. Materials and Methods

### 4.1. Subjects

In total, 17 RMS patients, regularly followed up at the Mondino Foundation in Pavia and diagnosed in accordance with the 2017 revised McDonald criteria [55], were enrolled in the study from September 2021 to June 2023. All participants aged 18 to 60 years were started on OCR treatment (T0) according to clinical practice [56]. Patients with recent MS relapse and/or those who had received a high dose of steroid treatment within a four-week period prior to inclusion, pregnant or lactating women, patients allergic to OCR, patients with a comorbidity (such as chronic disease of the immune system other than MS), and patients with active systemic infections were excluded. All recruited patients signed an informed consent form. A preliminary assessment of 17 healthy controls (HC), age and sex-matched, was also performed.

#### Clinical Data

The demographic, clinical, and radiological features of all enrolled MS patients were collected at the start of OCR treatment (T0) and after one year (T12). Specifically, detailed neurological and medical data were collected, with a focus on the presence and frequency of acute relapses, possible confirmed disability worsening, the annualized relapse rate (ARR), and disease duration. Disability and the severity of MS were evaluated with two scores: the Expanded Disability Status Scale (EDSS) [57] and the Multiple Sclerosis Severity Score (MSSS) [58], which were assigned according to the EDSS and MSSS scoring rules [58,59]. Further information included radiological (evaluation of signs of MRI activity) and safety information such as adverse events (AEs) and laboratory data (i.e., immunoglobulins levels, lymphocyte count). These data were collected at each assessment required by clinical practice (every six months).

### 4.2. PBMCs Isolation from Whole Blood

PBMCs from healthy donors and MS patients were isolated from peripheral blood before OCR treatment (T0) and after 12 months of exposure (T12). For each sample, drawn blood was diluted 1:1 with physiological solution (sodium chloride 0.9%), transferred into a 50 mL tube containing 15 mL of Lymphoprep^TM^ (Voden Medical Instruments Spa, Meda, Italy), and centrifuged at 800× *g* for 30 min (without brake). PBMCs were collected from the lymphocyte ring above the Lymphoprep^TM^ layer and washed twice with Dulbecco’s Phosphate Buffer Saline (PBS). Finally, after centrifugation at 500× *g* for 10 min, the supernatant was discarded, and the cellular pellets were resuspended in Fetal Bovine Serum (FBS) plus 10% DMSO and stored at −80 °C until further analysis.

### 4.3. Western Blotting

To evaluate PKCβII, HIF1α, VEGF, HuR, MnSOD, and HSP70, the PBMC samples were thawed and centrifuged at 500× *g* to obtain PBMC pellets. After that, the PBMCs were homogenized directly into a 2 mL tube by using a Pellet Pestle in a buffer containing 20 mM of Tris (pH 7.4), 2 mM of EDTA, 0.5 mM of EGTA and 50 mM of 2-β-mercaptoethanol, 0.32 M sucrose, with the addition of a protease inhibitor cocktail (Sigma-Aldrich, Darmstadt, Germany); this was sonicated 3 times for 20 s each time. The protein content of each sample was measured via the Bradford protein assay method, using bovine serum albumin (BSA; Sigma-Aldrich) as the standard. The proteins were diluted in Sodium Dodecyl Sulfate (SDS) protein gel loading solution 2X, boiled for 5 min at 95 °C, separated by 12% SDS-polyacrylamide gel electrophoresis (PAGE), and then transferred to nitrocellulose membranes (porosity: 0.2 μm) for 2 h at a constant electrical current of 250 mA. As a molecular weight marker, a standard mixture with colored proteins and a known molecular weight (Amersham, Chicago, CA, USA) was used. Unspecific sites were blocked with 6% milk in TBST buffer [10 mM of Tris-HCl, 100 mM of NaCl, 0.1% (*v*/*v*) Tween 20, pH 7.5] at room temperature for 1 h, and then the membranes were incubated with the primary antibodies overnight at 4 °C under gentle agitation. The anti-PKCβII (Santa Cruz Biotech, Santa Cruz, CA, USA) polyclonal antibody, the anti-HIF1α (Cell signaling Technology, Denver, CO, USA), and the anti-VEGF (Abcam, Cambridge, MA, USA) monoclonal antibodies were diluted 1:1000, 1:500, and 1:750, respectively, in TBST buffer containing 6% (*v*/*v*) milk, based on each data sheet. The anti-HuR (Santa Cruz Biotech), the anti-MnSOD (Santa Cruz Biotech), and the anti-HSP70 (Santa Cruz Biotech) monoclonal antibodies were diluted 1:1000, 1:200, and 1:1000, respectively. The next day, the membranes were washed and then incubated for 1 h with the secondary antibodies diluted according to the relative data sheet. The membrane signals were detected by chemiluminescence, employing an Imager Amersham 680 detection system and using ponceau (30–95 kDa) to normalize the data. The densitometric analysis of the western blots was performed using the ImageJ 1.54 image-processing program, after image acquisition.

### 4.4. Statistical Analysis

The clinical and demographic features of the MS patients were reported using either the mean and standard deviation (SD) for continuous variables or the median and interquartile (IQ) range for ordinal variables. Differences in the protein levels in various categories were assessed through non-parametric tests, i.e., the Mann–Whitney test for independent samples and the Wilcoxon signed-rank test for paired samples between T0 and T12. Normality was assessed through the plotting of values in a histogram and the visual inspection of the sample distribution, as normality tests were not deemed dependable when applied to the small size of our sample. The results are expressed as the mean grey level ratios × 10^3^ (mean ± S.E.M.) of the protein immunoreactivities measured by western blotting and normalized on ponceau signals. The correlation was assessed with Spearman’s correlation coefficient. A *p*-value below 0.05 was considered statistically significant. The statistical analysis was performed using GraphPad Prism statistical package (version 10 GraphPad software, San Diego, CA, USA).

## Figures and Tables

**Table 1:** Clinical and demographic features of patients affected by multiple sclerosis (MS) and healthy controls (HCs).

	MS Patients	Healthy Controls
	N = 17	N = 17
Age (years)	42.17 ± 8.44	42.00 ± 9.26
Sex (F/M ratio)	1.80	1.80
MS duration (years)	10.05 ± 9.26	
EDSS (N = 17)	2.79 ±1.70	
EDSS < 4	N 12 (70.6%)	
EDSS > 4	N 5 (29.4%)	
MSSS (N = 17)	4.24 ± 3.07	
Mild MS (MSSS < 3, N = 7)	1.12 ± 0.61	
Moderate/severe MS (MSSS ≥ 3, N = 10)	6.42 ± 1.90	

## Data Availability

The data presented in this study are available on request from the corresponding author. The data are not publicly available due to privacy.
